# Performance of different adiposity measures for predicting cardiovascular risk in adolescents

**DOI:** 10.1038/srep43686

**Published:** 2017-03-06

**Authors:** Min Zhao, Pascal Bovet, Chuanwei Ma, Bo Xi

**Affiliations:** 1 Department of Nutrition and Food Hygiene, School of Public Health, Shandong University, Jinan, China; 2 Institute of Social and Preventive Medicine, Lausanne University Hospital, Lausanne, Switzerland; 3 Department of Epidemiology, School of Public Health, Shandong University, Jinan, China

## Abstract

This study aims to compare the performance of body mass index (BMI), waist circumference (WC), and waist-to-height-ratio (WHtR) to predict the presence of at least 3 main CV risk factors in US adolescents. A total of 3621 adolescents (boys: 49.9%) aged 12–17 years from the US National Health and Nutrition Examination Survey (1999–2012) were included in this study. Measured CV risk factors included systolic/diastolic blood pressure, triglycerides, high-density lipoprotein cholesterol, low-density lipoprotein cholesterol, and fasting plasma glucose. The AUC of BMI-z score, WC-z score and WHtR-z score to predict at least three CV risk factors were similar (~0.85), irrespective of criteria used to define abnormal levels of CV risk factors. A 1-SD increase in any of three indices to predict CV risk was also similar for the three adiposity scores. For instance, a 1-SD increase risk in BMI-z score, WC-z score and WHtR-z score was 3.32 (95%CI 2.53–4.36), 3.43 (95%CI 2.64–4.46), and 3.45 (95%CI 2.64–4.52), respectively, in the total population using the International Diabetes Federation definition. In addition, the most efficient WHtR cut-off for screening CV risk was ~0.50 in US adolescents. In summary, BMI, WC and WHtR performed similarly well to predict the presence of at least 3 main CV risk factors among US adolescents.

The prevalence of obesity in children and adolescents increased greatly worldwide during the past few decades^1^. Pediatric obesity is associated with cardiovascular (CV) risk^2^ and target organ damage^3^ in childhood, as well as with CV disease and related premature mortality in middle or older ages^4^. Thus, obesity can be a useful simple index to identify children and adolescents at CV risk.

Body mass index (BMI) is a widely used to assess obesity worldwide. However, the use of BMI in children has several well known limitations. First, BMI cannot distinguish fat mass from lean mass, but BMI correlates strongly with both indicators. Second, BMI cut-offs in children varies according to age and sex, which makes it cumbersome to use in practice. Although waist circumference (WC) can potentially assess the distribution of fatness more accurately than BMI, it also requires age- and sex-specific cutoffs. The waist-to-height -ratio (WHtR) was recommended for assessing central obesity^5^. Although WHtR seems to be superior to WC and BMI for predicting diabetes and CV diseases in adults^6^,^7^, findings have been controversial in children and adolescents^8^,^9^,^10^,^11^,^12^,^13^,^14^,^15^,^16^,^17^,^18^,^19^,^20^,^21^. Several studies suggested that a WHtR cut-off of 0.50 performed best for assessing CV risk in children and adolescents^22^,^23^,^24^, but higher or lower cut-offs have also been suggested^16^,^17^,^25^,^26^. In addition, although WHtR is simple and convenient to use, it relies on measurement of waist, which accuracy depends substantially on the technique used by the observer and its measurement requests physical contact with the adolescents, which may be impractical in some settings and cultures.

In the present study, using data from the US National Health and Nutrition Examination Surveys (NHANES, 1999–2012), we aimed to assess which of three common obesity-related indices (BMI, WC and WHtR) performed best to identify CV risk. A second objective was to determine the optimal cut-off of WHtR in US adolescents.

## Methods

### Subjects

Data came from the NHANES in 1999–2012 (http://wwwn.cdc.gov/nchs/nhanes). NHANES uses a complex, multistage probability design to collect nationally representative sample of the US civilian, non-institutionalized population, which was conducted from 1999 to 2012 by the National Center for Health Statistics of the Centers for Disease Control and Prevention. NHANES is a program of studies designed to assess the health and nutritional status of adults and children in the United States, which includes household interviews and physical examinations in the mobile center. Data on demographics, questionnaire, examination, dietary and blood samples were collected. Our study was restricted to adolescents aged 12–17 years since fasting plasma glucose (FPG) was available only for subjects aged 12 years or older. A total of 3621 adolescents (boys: 49.9%) aged 12–17 years old were included (n = 665 in 1999–2000, n = 675 in 2001–2002, n = 634 in 2003–2004, n = 605 in 2005–2006, n = 308 in 2007–2008, n = 375 in 2009–2010, and n = 359 in 2011–2012), with complete data on sex, age, race, height, weight, BMI, WC, blood pressure (BP), triglycerides (TG), high-density lipoprotein cholesterol (HDL-C), low-density lipoprotein cholesterol (LDL-C), and FPG. Data on seven survey cycles between 1999–2000 and 2011–2012 were combined based on the NHANES analysis guideline 1999–2012 that new weights for combined data were computed as 2/7 multiplying by the original 4-year weights for surveys 1999–2002 and 1/7 multiplying by the original 2-year weights for surveys 2003–2012. All the procedures were conducted in accordance with the approved guidelines and relevant regulations. The NHANES was approved by the National Center for Health Statistics Ethics Review Board and informed consent from the study participants and their parents/guardians.

### Measurements

Measurements included height, weight, WC, systolic BP (SBP), diastolic BP (DBP), total cholesterol, TG, HDL-C, LDL-C and FPG levels^27^. Weight and height were measured for each individual in light clothing without shoes. BMI was calculated as weight (kg)/height squared (m^2^). WC was measured at the high point of the iliac crest^28^. WHtR was calculated as WC (cm)/height (cm).

SBP and DBP were measured using auscultatory mercury sphygmomanometers by trained examiners, following a standard protocol. BP was taken on the right arm with an appropriate cuff size (pediatric, normal or large cuffs) with the subject seating quietly for at least 5 minutes before BP measurement. The first phase (SBP) and the fifth phase (DBP) Korotkoff sounds were recorded. Data on DBP level as zero were excluded. Up to three BP measurements were taken for each individual and their average was used in the analyses.

Blood samples were obtained from the antecubital vein in at least 8-hour fasting subjects. TC, TG, HDL-C, and FPG were measured using enzymatic methods. LDL-C was estimated using the formula of Friedewald *et al*.^29^.

### Definition of CV risk factors

We defined abnormal levels of CV risk factors (BP, TG, HDL-C, and FPG) based on cut-offs used to define the metabolic syndrome (MetS) from the International Diabetes Federation (IDF)^30^ or the revised National Cholesterol Education Program Adult Treatment Panel III (ATP III) modified for the pediatric population^31^. One, or combination of two or three more CV risk factors was used as the outcomes. The IDF criteria in adolescents aged 12–15 years are defined as triglycerides ≥150 mg/dL (1.7 mmol/L), HDL-cholesterol <40 mgl/dL (1.03 mmol/L), SBP/DBP ≥130/85 mm Hg, and FPG ≥100 mg/dL (5.6 mmol/L) or known type 2 diabetes. Criteria for adults were applied for adolescents aged 16–17 years. The ATP III criteria are defined as triglycerides ≥110 mg/dL (1.24 mmol/L), HDL-C ≤40 mgl/dL (1.03 mmol/L), SBP/DBP ≥90^th^ percentile (age-, sex- and height specific), and FPG ≥110 mg/dL (6.1 mmol/L). High LDL-C was defined as LDL-C ≥100 mg/dL (2.6 mmol/L)^32^.

### Statistical analysis

All data analyses were performed using SAS 9.2 (SAS Institute, Cary, NC). Sampling weights, primary sampling unit (PSU) and strata were taken into consideration to account for the complex survey design. Quantitative data are expressed as mean [standard error (SE)] and categorical data as percentage (SE). Correlation coefficients between BMI, WC and WHtR were calculated using partial correlation analyses by sex adjusting for age and race/ethnicity. As BMI, WC and WHtR have different units, we calculated sex and age specific z-scores within the study sample. The performance of BMI-z score, WC-z score and WHtR-z score to predict the presence of one, or combination of two or three more from the five CV risk factors (BP, TG, HDL-C, LDL-C, and FPG), defined by either IDF or ATP III criteria, was assessed by comparing the area under curve (AUC) derived from receiver operator characteristic (ROC) curve analysis. ROC curve was constructed to visually show the relationship between true-positive (sensitivity) and false positive (1-specificity)^33^. The AUC was used to evaluate the performance of three adiposity indices in detecting CVD risk factors. An AUC = 0.5 indicated the test performed no better than chance. An AUC of <0.7 was considered as poor, 0.7–0.8 was rated as acceptable and of >0.8 as good. We performed logistic regression analysis to assess a cumulative CV risk associated with a 1-SD increase of adiposity measures. ROC analysis was also performed to determine the optimal WHtR cut-off. The optimal WHtR cut-off was determined by the maximum of the Youden index (sensitivity + specificity-1)^33^. This optimal threshold corresponds to the point that is closest to the top left-handed corner in the ROC curve with optimal sensitivity and specificity.

## Results

A total of 3621 adolescents (boys: 49.9%) aged 12–17 years were included in this study. Table 1 shows the characteristics and CV risk factors in boys and girls, respectively. The prevalence of clustering of CV risk factors differed between sexes and analyses were therefore also performed separately in boys and girls. In addition, BMI, WC and WHtR were highly correlated with each other (r ≥ 0.93 between either two indices, *p* < 0.001).

The AUC of BMI-z score, WC-z score, and WHtR-z score to predict at least three CV risk factors were virtually identical using IDF or ATP III criteria (Table 2). In the total population, AUC ranged from 0.84 to 0.85 for three obesity indices using either IDF or ATP III criteria, indicating good performance of all three indices. Similar results were obtained in sex and race/ethnicity subgroups (Table 2). The odds ratios associated with a 1-SD increase and the presence of at least three risk factors were largely similar for the three obesity indices (Table 2). In the total population, the odds ratios ranged from 3.32 to 3.45 using the IDF criteria and from 3.71 to 3.99 using the ATP III criteria. The results were largely independent of sex or race/ethnicity (Table 2). In addition, BMI-z score, WC-z score, and WHtR-z score also performed equally in predicting one or two CV risk factors based on ROC analysis and Logistic regression analysis (Supplemental Table 1).

The optimal cut-off to predict at least three CV risk factors was about 0.50 using either the IDF or ATP III criteria, with values ranging from 0.48 to 0.52 by sex and race/ethnicity (Table 3). In addition, ~0.50 as the cut-off of WHtR also performed well to predict one or two CV risk factors (Supplemental Table 2).

## Discussion

In the present study, we found that BMI, WHtR and WC performed similarly well to predict the presence of at least three CV risk factors in US adolescents. In addition, our findings confirm that the message “keep your waist less than half of your height” is valid in US adolescents. Indeed, the optimal WHtR cut-off was ~0.50 and was largely independent of sex, race/ethnicity, and criteria used to define CV risk factors.

Many studies have compared the performance of BMI, WC and WHtR for screening CV risk factors in children^8^,^9^,^10^,^11^,^12^,^13^,^14^,^15^,^16^,^17^,^18^,^19^,^20^. However, results have been inconsistent with some studies reporting that WHtR performed better than WC and BMI^8^,^9^,^10^,^11^,^14^, several studies reporting similar performance^16^,^17^,^18^,^19^, and others reporting that BMI performed best^20^,^21^. These conflicting results may be due to differences in study design, population selection, race/ethnicity, geographical location or methods used for assessing obesity-related indices. Our study is based on nationally representative data so that our findings can be generalized to all US adolescents. As there was no significant difference in using WHtR, WC and BMI for predicting CV risk, either obesity index are similarly valid for use in practice. However, because prediction based on WHtR only needs one value (0.50), this indicator may be more convenient in practice.

The optimal cut-off of WHtR for predicting CV risk has been debated and values of <0.50, 0.50 or >0.50 have been suggested^16^,^17^,^25^,^26^. An UK cohort study of 2710 children showed that 0.50 as WHtR cut-off had perfect specificity in predicting CV risk, but were poorly sensitive, suggesting that WHtR optimal cut-off should be less than 0.50^17^. In addition, a cross-sectional study conducted in South Africa reported that the cut-offs of 0.46 in boys and 0.45 in girls were optimal for screening CV risk^26^. In our study, a WHtR of 0.50 was most efficient in all US adolescents, independently of sex and tested race/ethnicity (Hispanic, White and Black). However, we have no WHtR data in other race/ethnicity such as Arabian and Asian. Thus, the optimal WHtR cut-off may differ in other populations and our conclusion should not be directly applied in Arabian or Asian. For instance, Asian children tend to be shorter than White or Black children for the same sex and age, and a lower WHtR cut-off has been suggested^34^.

Our study has several strengths. First, the sample of participants was nationally representative and our findings can be generalized to all US adolescents. Second, we assessed CV risk factors using different criteria and found that the results were independent of the criteria used to define CV risk factors. Third, measurements of CV risk factors followed strict quality control, including adequate calibration of instruments and training of the examiners. However, findings in our study should be validated in other countries and further studies should extend to children aged <12 years. In addition, the positive predictive value of WHtR (using 0.50 as optimal cut-off) for predicting 3 + CV risk factors was about 20% using IDF criteria and 25% using ATPIII because of low prevalence of clustering of 3 + CV risk factors, suggesting that WHtR alone has limited value to predict CV risk in adolescents.

## Conclusions

Our study suggests that BMI, WC. and WHtR performed well and almost identically for identifying the presence of at least 3 major CV risk factors in US adolescents and that a WHtR cut-off of ~0.50 is suitable for US adolescents. Our results were largely independent of sex and race/ethnicity. As the uses of BMI, WC or WHtR have each potential advantages and limitations in practice, translational research is needed to identify which index is most suitable in different screening and clinical conditions.

## Additional Information

**How to cite this article**: Zhao, M. *et al*. Performance of different adiposity measures for predicting cardiovascular risk in adolescents. *Sci. Rep.* **7**, 43686; doi: 10.1038/srep43686 (2017).

**Publisher's note:** Springer Nature remains neutral with regard to jurisdictional claims in published maps and institutional affiliations.

## Supplementary Material

Supplementary File

## Figures and Tables

**Table 1:** Characteristics of the US adolescents aged 12–17 years.

Characteristics	All (n = 3621)	Boys (n = 1868)	Girls (n = 1753)	*P* value#
Age, years	15.0 (0.1)	15.0 (0.1)	15.1 (0.1)	0.524
Weight, kg	63.2 (0.4)	65.8 (0.6)	60.5 (0.6)	<0.001
Height, cm	165.5 (0.2)	169.4 (0.4)	161.5 (0.3)	<0.001
BMI, kg/m^2^	22.9 (0.1)	22.7 (0.2)	23.1 (0.2)	0.124
WC, cm	79.8 (0.3)	79.8 (0.5)	79.7 (0.5)	0.924
SBP, mmHg	108.5 (0.3)	111.0 (0.4)	105.9 (0.3)	<0.001
DBP, mmHg	60.5 (0.4)	59.4 (0.4)	61.8 (0.5)	<0.001
TG, mg/dl	84.7 (1.4)	86.6 (2.4)	82.9 (1.6)	0.194
HDL-C, mg/dl	51.8 (0.3)	50.0 (0.4)	53.6 (0.4)	<0.001
FPG, mg/dl	93.2 (0.3)	95.2 (0.5)	91.2 (0.3)	<0.001
LDL-C, mg/dl	89.1 (0.5)	88.7 (1.0)	89.5 (0.7)	<0.001
**Race/ethnicity**				0.408
Mexican American	1143 (11.7)	570 (12.2)	573 (11.2)	
Other Hispanic	214 (5.7)	112 (6.0)	102 (5.4)	
Non-Hispanic White	993 (61.6)	502 (60.0)	491 (63.2)	
Non-Hispanic Black	1053 (14.2)	570 (14.2)	483 (14.2)	
Others	218 (6.8)	114 (7.6)	104 (6.0)	
**CV risk factors**				
IDF criteria*				
High BP, %	109 (2.8)	92 (4.9)	17 (0.7)	<0.001
High TG, %	271 (7.7)	151 (8.4)	120 (7.0)	0.248
Low HDL-C, %	642 (17.9)	315 (17.7)	327 (18.1)	0.789
High FPG, %	552 (16.0)	389 (21.6)	163 (10.4)	<0.001
High LDL-C, %	1080 (28.9)	537 (27.4)	543 (30.3)	0.207
ATPIII criteria*				
High BP, %	556 (14.4)	411 (21.2)	145 (7.6)	<0.001
High TG, %	687 (19.8)	378 (21.6)	309 (18.0)	0.055
Low HDL, %	578 (16.5)	358 (20.1)	220 (12.9)	<0.001
High FPG, %	62 (1.6)	41 (1.9)	21 (1.2)	0.162
High LDL-C, %	1080 (28.9)	537 (27.4)	543 (30.3)	0.207
**CV risk factor clustering**				
IDF criteria*				0.019
None	1746 (48.5)	870 (46.4)	876 (50.6)	
1	1274 (35.5)	634 (35.2)	640 (35.7)	
2	451 (11.9)	267 (13.1)	184 (10.8)	
≥3	140 (4.1)	91 (5.4)	49 (2.9)	
ATPIII criteria*				<0.001
None	1714 (48.3)	816 (44.4)	898 (52.2)	
1	1138 (30.6)	582 (31.2)	556 (30.0)	
2	522 (14.5)	300 (15.0)	222 (13.9)	
≥3	237 (6.6)	164 (9.3)	73 (3.9)	

BMI, body mass index; WC, waist circumference; SBP, systolic blood pressure; DBP, diastolic blood pressure; TG, triglyceride; HDL-C, high density lipoprotein-cholesterol; FPG, fasting plasma glucose; IDF, International Diabetes Federation; ATP III, Adult Treatment Panel III.

Data are expressed as mean and standard error (SE) or n (%).

^*^Exclusion of WC and inclusion of LDL-C.

^#^Difference between boys and girls, *t* test for continuous variables and Chi-squared test for category variables.

**Table 2:** Performance of BMI, WC and WHtR to predict the presence of at least 3 cardiovascular risk factors.

Groups	No. of subjects	AUC (95% CI)*			OR (95%CI)#		
		BMI-Z score	WC-Z score	WHtR-Z score	BMI-Z score	WC-Z score	WHtR-Z score
**IDF criteria**							
Total	3621	0.84 (0.80–0.87)	0.84 (0.81–0.88)	0.85 (0.82–0.88)	3.32 (2.53–4.36)	3.43 (2.64–4.46)	3.45 (2.64–4.52)
Boys	1868	0.80 (0.76–0.85)	0.81 (0.77–0.86)	0.82 (0.78–0.86)	4.50 (3.18–6.36)	4.48 (3.29–6.10)	4.48 (3.26–6.17)
Girls	1753	0.81 (0.74–0.87)	0.81 (0.75–0.87)	0.81 (0.75–0.87)	2.38 (1.53–3.69)	2.41 (1.53–3.82)	2.48 (1.56–3.95)
Hispanic	1357	0.86 (0.81–0.91)	0.86 (0.81–0.91)	0.86 (0.81–0.91)	4.91 (3.43–7.01)	4.72 (3.48–6.39)	4.33 (3.27–5.73)
White	993	0.82 (0.74–0.89)	0.82 (0.75–0.90)	0.83 (0.76–0.90)	3.32 (2.25–4.90)	3.40 (2.32–4.97)	3.48 (2.37–5.11)
Black	1053	0.83 (0.75–0.92)	0.83 (0.74–0.93)	0.85 (0.78–0.92)	2.29 (1.65–3.18)	2.56 (1.84–3.57)	2.51 (1.90–3.31)
**ATPIII criteria**							
Total	3621	0.85 (0.82–0.87)	0.84 (0.81–0.87)	0.85 (0.83–0.88)	3.72 (2.94–4.70)	3.71 (2.99–4.60)	3.99 (3.20–4.97)
Boys	1868	0.86 (0.83–0.89)	0.85 (0.81–0.89)	0.86 (0.83–0.90)	4.46 (3.09–6.46)	4.26 (3.09–5.87)	4.80 (3.50–6.58)
Girls	1753	0.84 (0.80–0.89)	0.84 (0.79–0.89)	0.85 (0.81–0.89)	3.00 (2.21–4.05)	3.09 (2.29–4.18)	3.17 (2.30–4.38)
Hispanic	1357	0.87 (0.83–0.91)	0.86 (0.82–0.91)	0.88 (0.84–0.91)	4.85 (3.55–6.63)	4.92 (3.67–6.59)	4.91 (3.62–6.65)
White	993	0.85 (0.80–0.90)	0.85 (0.79–0.90)	0.86 (0.81–0.91)	4.04 (2.84–5.75)	3.91 (2.82–5.42)	4.20 (3.03–5.81)
Black	1053	0.81 (0.74–0.87)	0.80 (0.74–0.87)	0.82 (0.76–0.87)	2.36 (1.79–3.10)	2.39 (1.78–3.22)	2.49 (1.88–3.30)

AUC, area under the curve in ROC analysis; CI, confidence interval; OR, odds ratio; IDF, International Diabetes Federation; ATP III, Adult Treatment Panel III.

^*^Receiver operator characteristic curve analysis.

^#^1-SD increase using logistic regression analysis with adjustment for sex, age and race where appropriate.

**Table 3:** Optimal cut-offs of WHtR to predict the presence of at least 3 cardiovascular risk factors.

Groups	No. of subjects	Optimal cut-offs	AUC (95% CI)	Sensitivity	Specificity
**IDF criteria**					
Total	3621	0.52	0.85 (0.81–0.88)	0.72	0.84
Boys	1868	0.48	0.88 (0.85–0.92)	0.86	0.79
Girls	1753	0.51	0.81 (0.74–0.87)	0.76	0.76
Hispanic	1357	0.51	0.85 (0.80–0.91)	0.84	0.77
White	993	0.50	0.83 (0.76–0.90)	0.74	0.82
Black	1053	0.48	0.85 (0.77–0.91)	0.86	0.75
**ATPIII criteria**					
Total	3621	0.51	0.84 (0.81–0.87)	0.75	0.83
Boys	1868	0.49	0.87 (0.84–0.90)	0.79	0.84
Girls	1753	0.52	0.85 (0.80–0.89)	0.81	0.77
Hispanic	1357	0.51	0.87 (0.83–0.91)	0.83	0.79
White	993	0.51	0.85 (0.79–0.90)	0.76	0.86
Black	1053	0.48	0.80 (0.74–0.86)	0.76	0.73

AUC, area under the curve in ROC analysis; CI, confidence interval; IDF, International Diabetes Federation; ATP III, Adult Treatment Panel III.
