# Supplementary material for: Performance of different adiposity measures for predicting cardiovascular risk in adolescents
Source: Sci Rep. 2017 Mar 6;7:43686. doi: 10.1038/srep43686 (PMC5337956; doi:10.1038/srep43686)
Supplement: Supplementary File [file srep43686-s1.doc]

**Supplementary information**

**Performance of different adiposity measures for predicting cardiovascular risk in adolescents**

Min Zhao, MD ^1^ Pascal Bovet, MD ^2^ Chuanwei Ma, MS ^3^ Bo Xi, MD ^3*^

1. Department of Nutrition and Food Hygiene, School of Public Health, Shandong University, Jinan, China
2. Institute of Social and Preventive Medicine, Lausanne University Hospital, Lausanne, Switzerland
3. Department of Epidemiology, School of Public Health, Shandong University, Jinan, China

*** Corresponding author:**

Bo Xi, 44 Wenhua Xi Road, Department of Epidemiology, School of Public Health, Shandong University, Jinan 250012, China. Tel/Fax: 86-531-88382141; Email: xibo2007@126.com

**Running title:** Performance of adiposity measures

**Supplemental Table 1 Performance of BMI, WC and WHtR to predict the presence of one or two more** cardiovascular risk factors

| Groups | No. of subjects | AUC (95% CI) ^*^ | | | OR (95%CI) ^#^ | | |
| --- | --- | --- | --- | --- | --- | --- | --- |
|  |  | BMI-Z score | WC-Z score | WHtR-Z score | BMI-Z score | WC-Z score | WHtR-Z score |
| **IDF criteria** |  |  |  |  |  |  |  |
| **>=1 CV risk factors** |  |  |  |  |  |  |  |
| Total | 3621 | 0.64 (0.62-0.65) | 0.64 (0.62-0.66) | 0.64 (0.63-0.66) | 1.61 (1.41-1.83) | 1.61 (1.42-1.84) | 1.69 (1.48-1.93) |
| Boys | 1868 | 0.65 (0.63-0.68) | 0.66 (0.63-0.68) | 0.67 (0.64-0.69) | 1.82 (0.54-2.15) | 1.88 (1.60-2.19) | 1.97 (1.68-2.32) |
| Girls | 1753 | 0.62 (0.59-0.65) | 0.62 (0.59-0.65) | 0.62 (0.59-0.65) | 1.44 (1.20-1.72) | 1.42 (1.18-1.70) | 1.48 (1.24-1.78) |
| Hispanic | 1357 | 0.68 (0.65-0.71) | 0.68 (0.65-0.71) | 0.68 (0.65-0.71) | 2.30 (1.96-2.69) | 2.32 (1.99-2.70) | 2.28 (1.96-2.65) |
| White | 993 | 0.60 (0.56-0.64) | 0.60 (0.57-0.64) | 0.61 (0.58-0.65) | 1.45 (1.22-1.76) | 1.47 (1.23-1.75) | 1.56 (1.30-1.87) |
| Black | 1053 | 0.63 (0.59-0.66) | 0.63 (0.59-0.66) | 0.65 (0.61-0.68) | 1.57 (1.40-1.76) | 1.60 (1.42-1.80) | 1.64 (1.15-1.87) |
| **>=2 CV risk factors** |  |  |  |  |  |  |  |
| Total | 3621 | 0.74 (0.71-0.76) | 0.75 (0.73-0.77) | 0.76 (0.74-0.78) | 2.23 (1.91-2.61) | 2.29 (1.97-2.66) | 2.43 (2.08-2.83) |
| Boys | 1868 | 0.76 (0.73-0.79) | 0.77 (0.74-0.81) | 0.79 (0.76-0.82) | 2.83 (2.27-3.53) | 3.03 (2.45-3.75) | 3.25 (2.60-4.08) |
| Girls | 1753 | 0.71 (0.67-0.75) | 0.72 (0.68-0.76) | 0.72 (0.68-0.76) | 1.76 (1.42-2.20) | 1.74 (1.41-2.16) | 1.84 (1.49-2.27) |
| Hispanic | 1357 | 0.78 (0.75-0.82) | 0.78 (0.75-0.82) | 0.79 (0.75-0.82) | 3.36 (2.68-4.22) | 3.45 (2.71-4.40) | 3.41 (2.68-4.35) |
| White | 993 | 0.69 (0.64-0.74) | 0.70 (0.65-0.75) | 0.72 (0.67-0.77) | 2.02 (1.65-2.46) | 2.03 (1.70-2.44) | 2.20 (1.81-2.67) |
| Black | 1053 | 0.73 (0.68-0.79) | 0.75 (0.70-0.80) | 0.76 (0.71-0.81) | 2.17 (1.84-2.55) | 2.30 (1.93-2.75) | 2.36 (1.98-2.82) |
| **ATPIII criteria** |  |  |  |  |  |  |  |
| **>=1 CV risk factors** |  |  |  |  |  |  |  |
| Total | 3621 | 0.65 (0.63-0.67) | 0.65 (0.63-0.66) | 0.65 (0.63-0.67) | 1.74 (1.55-1.95) | 1.70 (1.51-1.92) | 1.81 (1.62-2.03) |
| Boys | 1868 | 0.67 (0.65-0.69) | 0.67 (0.64-0.69) | 0.67 (0.65-0.70) | 1.97 (1.62-2.39) | 1.97 (1.63-2.38) | 2.13 (1.76-2.58) |
| Girls | 1753 | 0.64 (0.61-0.66) | 0.63 (0.60-0.66) | 0.63 (0.61-0.66) | 1.56 (1.36-1.79) | 1.51 (1.31-1.75) | 1.60 (1.39-1.84) |
| Hispanic | 1357 | 0.70 (0.67-0.73) | 0.70 (0.67-0.73) | 0.70 (0.67-0.73) | 2.18 (1.81-2.62) | 2.20 (1.82-2.66) | 2.17 (1.79-2.62) |
| White | 993 | 0.62 (0.58-0.65) | 0.61 (0.58-0.65) | 0.63 (0.59-0.66) | 1.65 (1.39-1.95) | 1.61 (1.36-1.89) | 1.76 (1.50-2.07) |
| Black | 1053 | 0.63 (0.60-0.66) | 0.63 (0.60-0.66) | 0.64 (0.61-0.67) | 1.54 (1.36-1.75) | 1.55 (1.36-1.77) | 1.59 (1.39-1.83) |
| **>=2 CV risk factors** |  |  |  |  |  |  |  |
| Total | 3621 | 0.75 (0.72-0.77) | 0.75 (0.73-0.77) | 0.76 (0.74-0.78) | 2.26 (1.97-2.59) | 2.35 (2.04-2.71) | 2.51 (2.20-2.88) |
| Boys | 1868 | 0.77 (0.74-0.80) | 0.78 (0.75-0.80) | 0.79 (0.76-0.82) | 2.83 (2.27-3.54) | 3.02 (2.40-3.79) | 3.30 (2.63-4.15) |
| Girls | 1753 | 0.72 (0.68-0.75) | 0.72 (0.69-0.76) | 0.73 (0.70-0.76) | 1.82 (1.51-2.20) | 1.89 (1.57-2.26) | 1.99 (1.66-2.38) |
| Hispanic | 1357 | 0.80 (0.77-0.83) | 0.80 (0.77-0.83) | 0.81 (0.78-0.84) | 3.45 (2.79-4.27) | 3.47 (2.74-4.39) | 3.61 (2.83-4.60) |
| White | 993 | 0.68 (0.64-0.72) | 0.69 (0.65-0.74) | 0.71 (0.66-0.75) | 2.06 (1.71-2.47) | 2.14 (1.79-2.56) | 2.33 (1.95-2.79) |
| Black | 1053 | 0.74 (0.69-0.78) | 0.74 (0.69-0.78) | 0.75 (0.70-0.79) | 2.15 (1.77-2.61) | 2.20 (1.83-2.65) | 2.28 (1.88-2.77) |

AUC, area under the curve in ROC analysis; CI, confidence interval; OR, odds ratio; IDF, International Diabetes Federation; ATP III, Adult Treatment Panel III

^*^ Receiver operator characteristic curve analysis

^#^ 1-SD increase using logistic regression analysis with adjustment for sex, age and race where appropriate

**Supplemental Table 2. Optimal cut-offs of WHtR to predict the presence of one or two more** cardiovascular risk factors

| Groups | No. of subjects | Optimal cut-offs | AUC (95% CI) | Sensitivity | Specificity |
| --- | --- | --- | --- | --- | --- |
| **IDF criteria** |  |  |  |  |  |
| **>=1 CV risk factors** |  |  |  |  |  |
| Total | 3621 | 0.48 | 0.64 (0.62-0.66) | 0.53 | 0.70 |
| Boys | 1868 | 0.48 | 0.67 (0.64-0.69) | 0.51 | 0.78 |
| Girls | 1753 | 0.49 | 0.62 (0.60-0.65) | 0.55 | 0.66 |
| Hispanic | 1357 | 0.49 | 0.67 (0.65-0.70) | 0.61 | 0.70 |
| White | 993 | 0.48 | 0.61 (0.57-0.64) | 0.51 | 0.68 |
| Black | 1053 | 0.47 | 0.65 (0.62-0.68) | 0.59 | 0.66 |
| **>=2 CV risk factors** |  |  |  |  |  |
| Total | 3621 | 0.49 | 0.75 (0.73-0.78) | 0.67 | 0.75 |
| Boys | 1868 | 0.48 | 0.79 (0.76-0.82) | 0.70 | 0.79 |
| Girls | 1753 | 0.49 | 0.72 (0.69-0.76) | 0.70 | 0.67 |
| Hispanic | 1357 | 0.49 | 0.78 (0.74-0.81) | 0.78 | 0.70 |
| White | 993 | 0.48 | 0.71 (0.67-0.76) | 0.63 | 0.71 |
| Black | 1053 | 0.48 | 0.76 (0.71-0.81) | 0.67 | 0.77 |
| **ATPIII criteria** |  |  |  |  |  |
| **>=1 CV risk factors** |  |  |  |  |  |
| Total | 3621 | 0.49 | 0.64 (0.63-0.66) | 0.51 | 0.75 |
| Boys | 1868 | 0.48 | 0.68 (0.65-0.70) | 0.52 | 0.78 |
| Girls | 1753 | 0.49 | 0.63 (0.60-0.66) | 0.56 | 0.67 |
| Hispanic | 1357 | 0.49 | 0.69 (0.66-0.72) | 0.62 | 0.71 |
| White | 993 | 0.48 | 0.62 (0.59-0.65) | 0.51 | 0.70 |
| Black | 1053 | 0.47 | 0.64 (0.60-0.67) | 0.58 | 0.65 |
| **>=2 CV risk factors** |  |  |  |  |  |
| Total | 3621 | 0.49 | 0.75 (0.73-0.77) | 0.65 | 0.76 |
| Boys | 1868 | 0.48 | 0.79 (0.76-0.82) | 0.69 | 0.82 |
| Girls | 1753 | 0.51 | 0.73 (0.69-0.76) | 0.61 | 0.77 |
| Hispanic | 1357 | 0.49 | 0.79 (0.76-0.82) | 0.77 | 0.71 |
| White | 993 | 0.48 | 0.70 (0.66-0.74) | 0.61 | 0.73 |
| Black | 1053 | 0.48 | 0.74 (0.69-0.78) | 0.67 | 0.74 |

AUC, area under the curve in ROC analysis; CI, confidence interval; IDF, International Diabetes Federation; ATP III, Adult Treatment Panel III
